# Facile Construction of Functionalized GO Nanocomposites with Enhanced Antibacterial Activity

**DOI:** 10.3390/nano9070913

**Published:** 2019-06-26

**Authors:** Lei Jiang, Zhongjie Zhu, Yanyi Wen, Shan Ye, Chen Su, Rui Zhang, Wei Shao

**Affiliations:** 1 Jiangsu Co-Innovation Center of Efficient Processing and Utilization of Forest Resources, Nanjing Forestry University, Nanjing 210037, China; 2 College of Chemical Engineering, Nanjing Forestry University, Nanjing 210037, China

**Keywords:** graphene, polyethyleneimine, controlled release, antibacterial

## Abstract

The development of antimicrobial materials with sustained drug release performance is of great importance. Graphene oxide (GO) is considered to be an ideal drug carrier. In this study, tetracycline hydrochloride (TC) was loaded onto polyethyleneimine-functionalized GO (PG) to fabricate TC/PG nanocomposites. The success of the fabrication was confirmed by zeta potential, TEM, FTIR, and Raman analyses. The TC/PG nanocomposites showed a controlled and sustained drug release behavior, and a pseudo second order kinetic model was employed to illustrate the release mechanism. The antibacterial activity was studied using the disk diffusion method against *Escherichia coli* and *Staphylococcus aureus*. The TC/PG nanocomposites exhibited great bacterial inhibition performance. The results indicate that the fabricated TC/PG nanocomposites with effective antibacterial activity have great potential in antibacterial applications.

## 1. Introduction

Graphene, a two-dimensional (2D) carbon material that is one atom thick, has attracted the attention of researchers in various fields due to its exceptional optical, electronic, thermal, and mechanical properties [1,2,3,4]. Graphene oxide (GO) is a derivative of graphene and owns many functional groups, including epoxide, hydroxyl, carboxyl, and carbonyl groups [5]. GO has great potential in biomedical applications due to its high surface area, good biocompatibility, antibacterial properties, and capacity for facile functionalization using both chemical and biological methods [6]. GO has attracted attention in drug-delivery and controlled-release applications due to its intrinsic conjugated π-structure and ease of functionalization [7]. Nanosized fluorinated GO sheets for a targeted drug delivery system with switchable fluorescence, high near-infrared absorption, and controlled drug release performance were constructed in [8]. Poly(amidoamine)-modified GO as a siRNA vector was developed and exhibited great potential in gene-based antitumor therapy due to its pH-triggered release performance [9]. A chlorogenic acid–GO nanocomposite with sustained and pH-dependent release performance was successfully synthesized and exhibited an insignificant toxicity effect towards a normal cell line and an enhanced toxic effect towards the evaluated cancer cell lines [10].

In recent years, the antibacterial performance of GO nanosheets has been widely studied, and several antibacterial mechanisms have been proposed, including nanoknives, trapping or wrapping, and oxidative stress [11,12,13]. The physicochemical properties of GO, including its shape, dimensions, and surface functionalization, can influence its antibacterial behavior [14]. GO with sharp edges can easily permeate into a bacterial membrane due to this unique structure [15]. GO with a larger lateral size has been reported to exhibit a stronger bacteria killing ability [16]. However, in another study, GO of a smaller size was reported to exhibit higher antibacterial activity than GO of a larger size [17]. The functionalization of GO enriches graphene-based nanomaterial applications, which range from electrical conductivity to antibacterial use [18]. GO/quaternary ammonium salt nanocomposites were successfully fabricated by non-covalent functionalization and showed controlled drug release and good antibacterial performance [19]. Poly[5,5-dimethyl-3-(3′-triethoxysilylpropyl)hydantoin]/GO nanocomposites were synthesized through covalent functionalization and exhibited great antibacterial activity [20]. A UiO-66/GO membrane was fabricated and shown to possess good antimicrobial activity that would effectively prevent biofouling [21]. Moreover, metal and metal-oxide nanoparticles were loaded onto GO nanosheets to endow them with ideal antibacterial performance [22,23,24]. Silver-doped laser-induced graphene was shown to exhibit potent surface antibacterial and anti-biofilm activity [25]. A novel copper nanoparticles-decorated graphene sponge was synthesized successfully and could be used as a novel bactericidal filter due to its high antibacterial activity [26]. GO nanosheets were applied as drug carriers for antibiotics in order to achieve outstanding synergistic antibacterial effects [27].

Polyethyleneimine (PEI) is considered to be an ideal surface modifier due to its intrinsic polycationic performance with a high charge density and a high isoelectric point (>10) [1]. Therefore, it is believed that the fabrication of PEI-functionalized GO (PG) nanosheets will endow GO with a high positive surface charge and increased stability. These PG nanosheets with a positive charge could easily adsorb onto negatively charged bacterial cell membranes, giving the fabricated PG nanosheets enhanced antibacterial activity. The formation of electrostatic interactions, hydrogen bonds, and covalent bonds between PEI and GO provide PG nanosheets with a supramaximal specific surface area, which makes PG nanosheets an ideal drug carrier [28]. PEI-functionalized GO was able to deliver DNA effectively, suggesting that it is an ideal candidate for gene delivery [29,30]. Photothermal, silver-loaded, PEI-mediated magnetic GO composites were successfully prepared in [31]. In our previous study, PEI was successfully covalently linked to GO using EDC·HCl as the catalyst, and it exhibited a synergistic antibacterial effect [32].

In order to develop new antibacterial agents with highly reduced doses of antibiotics for combating micro-organisms and suppressing bacterial activity, tetracycline hydrochloride (TC), which has broad-spectrum antibacterial activity, was loaded onto PG to produce TC/PG nanocomposites. In this study, PEI was used to functionalize GO without a catalyst. The surface morphology and hydrophilicity were studied. The TC release behavior and antibacterial performance of the TC/PG nanocomposites were evaluated.

## 2. Materials and Methods

### 2.1. Fabrication

One gram (1 g) of PEI (M_w_ = 70,000, 50%) was added to a 2 mg/mL 50 mL GO suspension (XFNANO Materials Tech Co. Ltd., Nanjing, China) and stirred for 24 h. The suspension was centrifugated at 12,000× *g* rpm for 10 min, rinsed with de-ionized water, and freeze-dried. A 20 mL mixture of PG and TC was prepared with a fixed concentration (10% *w*/*w*). Then, the mixture was filtered through a cellulose acetate membrane with a 0.22 µm pore size. Finally, TC/PG nanocomposites were obtained using the freeze-dry method.

### 2.2. Characterization

Transmission electron microscopy (TEM) pictures were obtained with a JEM-2100 TEM instrument at an accelerating voltage of 200 kV. Fourier-transform infrared (FTIR) analysis was carried out on a Spectrum Two FTIR Spectrometer (Perkin Elmer, Akron, OH, USA). Raman spectra were obtained on a DXR Smart Raman spectrometer (Thermo Fisher Scientific, Waltham, MA, USA) using a laser excitation of 532 nm.

### 2.3. Release Behavior

Five milligrams (5 mg) of nanocomposite was placed into a 3500 Da dialysis bag, immersed in 50 mL of phosphate-buffered saline (PBS) buffer (pH = 7.4), and sealed. Then, 2.5 mL of solution was taken out at fixed time intervals and tested at a wavelength of 356 nm using a SHIMADZU 2450 UV spectrophotometer (Shimadzu Corporation, Kyoto, Japan). Finally, 2.5 mL of fresh PBS buffer solution was added into the system, and the concentration of released TC was measured.

### 2.4. Antibacterial Activity

#### 2.4.1. Inhibition of Growth Effect

One hundred microliters (100 µL) of pre-cultured *Escherichia coli* and *Staphylococcus aureus* suspensions with a concentration of 1 × 10^6^ colony-forming units (CFU)/mL were added into 40 mL of Tryptone Soya Broth (TSB) medium, respectively. Then, different amounts of TC/PG nanocomposites were added to achieve concentrations of 0.5, 1, 2, 4, and 8 µg/mL. The mixtures were incubated at 37 °C for 8 h at 150 rpm. Then, 2.5 mL of supernatant was taken out and tested at a wavelength of 600 nm (optical density (OD)) using a UV spectrophotometer (SHIMADZU 2450). The inhibition activity (*I*, *%*) was calculated as follows:

(1) $$I(\%)=\frac{OD_{control}-OD_{sample}}{OD_{control}}\times 100\%$$

where *OD_control_* is the OD value at 600 nm of the bacterial supernatant without any treatment as the control, and *OD_sample_* is the OD value at 600 nm of the bacterial supernatant with treatment with different samples, respectively.

Furthermore, the supernatant was diluted to 10^−3^ times the concentration, dispersed onto TSB plates, and incubated at 37 °C for 24 h. The number of surviving micro-organisms was observed and pictures were taken.

#### 2.4.2. Fluorescent Stain

One milligram (1 mg) of nanocomposite was added to 20 mL *E. coli* and *S. aureus* suspensions containing 1 × 10^6^ CFU/mL cells, respectively. The suspensions were incubated at 120 rpm at 37 °C for 2 h, followed by staining with propidium iodide (PI) and SYTO9 for 15 min under dark conditions, respectively. Then, they were imaged by an Olympus Fluorescence Microscope (IX53, Olympus, Tokyo, Japan). The bacterial suspension without treatment was used as the control.

#### 2.4.3. Inhibition Zone Behavior

A TC/PG nanocomposite-loaded paper was cut into round shapes with a diameter of 10 mm, and an ultraviolet lamp was applied for 60 min to sterilize them. Lawns of *E. coli* and *S. aureus* with about 1 × 10^5^ CFU/plate on the Tryptone Soya Agar (TSA) were pre-prepared. The sterilized specimens were placed upon them carefully. Then, the plates were incubated at 37 °C for one day. The bacterial inhibition ability was estimated by measuring the inhibition zone diameter.

#### 2.4.4. Bacterial Morphologies

Twenty microliters (20 µL) of bacterial suspension was added to 40 mL of TSB and incubated at 120 rpm at 37 °C for 4 h. Then, 1 mg of TC/PG nanocomposite was added. The bacteria with and without nanocomposite treatment were fixed using 4% glutaraldehyde for 24 h. The specimens were dehydrated with sequential ethanol treatments for 10 min and lyophilized. They were fixed on an aluminum stub, coated with platinum, and observed by a JSM-7600F SEM (JEOL, Tokyo, Japan).

#### 2.4.5. Protein Leakage

The pre-incubated *E. coli* and *S. aureus* suspensions were centrifugated at 6000 rpm for 5 min, rinsed three times with 0.9% NaCl, and diluted in 0.9% NaCl to obtain OD_600_ values of 1.5. Two hundred microliters (200 μL) of TC/PG nanocomposite (1 mg/mL) was put in 20 mL of bacterial suspension. The control group without any sample was used. The above mixture was incubated at 120 rpm at 37 °C for 4 h. Forty microliters (40 µL) of supernatant was put into 400 µL of working solution, which was obtained using an Enhanced BCA Protein Assay Kit (Beyotime, Shanghai, China). After incubation at 37 °C for 30 min, the absorption of the above solution was tested at a wavelength of 562 nm. The protein leakage concentrations were calculated.

#### 2.4.6. Detection of Reactive Oxygen Species (ROS) Production

Oxidative stress measurements were carried out with 2,3-bis(2-methoxy-4-nitro-5-sulfophenyl)-2H-tetrazolium-5-carboxanilide (XTT, Invitrogen, Eugene, OR, USA) and Ellman’s assays. XTT was added into a 0.01 M PBS buffer solution to obtain a concentration of 0.4 mM. One hundred microliters (100 μL) of a 2 mg/mL TC/PG nanocomposite dispersion was put in 2 mL of XTT solution and incubated for 2–6 h in the dark at 150 rpm. The mixture was filtered (0.22 μm), and the supernatant was tested at a wavelength of 470 nm using a Spectrum Two UV Spectrometer. XTT solution without any sample was used as the control. Reduced glutathione (GSH) oxidation was measured using an Ellman’s assay [33]. Briefly, GSH was added into 50 mM bicarbonate buffer (pH 8.6) to obtain a concentration of 0.8 mM. One hundred microliters (100 μL) of TC/PG nanocomposite dispersion (2 mg/mL) was put in 2 mL of GSH bicarbonate buffer. It was incubated for 2–6 h in the dark at 150 rpm. Then, 1.5 mL of 50 mM Tris-HCl and 30 μL of 100 mM DNTB (Molecular Probe) solution was put in the above mixture, and a product exhibiting a yellow color was formed. It was filtered (0.22 μm), and the supernatant was tested at a wavelength of 412 nm using a Spectrum Two UV Spectrometer. GSH solution without any sample was applied as the negative control, and 1 mM H_2_O_2_ was put in 0.4 mM GSH solution as the positive control. The GSH loss percentage (L_G_) was determined using the following equation:

(2) $$L_{G}=\frac{A_{n}-A_{s}}{A_{n}}\times 100\%$$

where *A_n_* and *A_s_* are the absorption values of the negative control and samples, respectively.

### 2.5. Statistical Analysis

The OriginPro 8 software was used to analyze the data, which are expressed as mean ± standard error (SE). Statistical differences were evaluated using a Student’s *t*-test. A *p*-value of <0.05 was considered to be statistically significant.

## 3. Results and Discussion

### 3.1. Morphology

**Figure 1:**
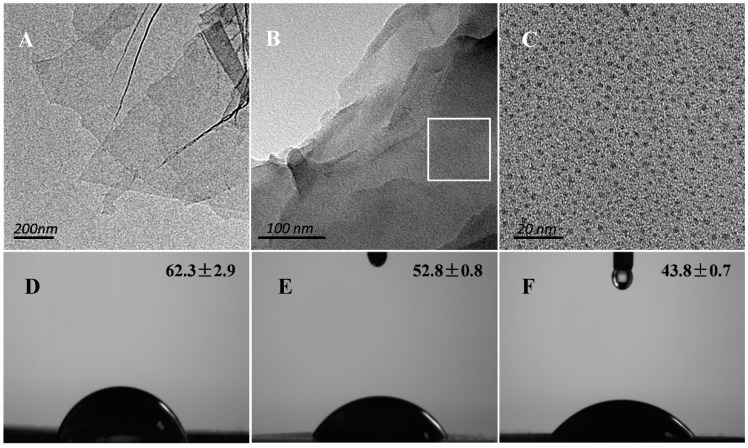
TEM images of graphene oxide (GO) (**A**) and tetracycline hydrochloride (TC)/polyethyleneimine (PEI)-functionalized GO (PG) (**B**,**C**). C shows a higher magnification of the square location in image B. Contact angle pictures of GO (**D**), PG (**E**), and TC/PG (**F**).

TEM images of GO and TC/PG nanocomposites are displayed in Figure 1. As shown in Figure 1A, the GO nanosheet exhibits an almost transparent single layer, which is consistent with the previous report [34]. Figure 1B shows the morphology of the TC/PG nanocomposite. Its transparency is clearly reduced after PEI functionalization. Figure 1C is an enlarged image of the square location on the TC/PG nanocomposite shown in Figure 1B. It can be seen that the TC has spherical and dark spots, and is dispersed on the PG nanosheets uniformly, indicating that TC was successfully loaded onto the functionalized GO nanosheets. The Zeta potentials of the GO nanosheets, the PG nanosheets, and the TC/PG nanocomposites were tested. The GO nanosheets carry a negative charge, and the measured Zeta potential of the 2 mg/mL GO nanosheets is −65.5 ± 1.2 mV. The high negative charges can make it difficult for the GO nanosheets to approach the bacterial cell membrane due to the existence of a repulsive interaction. Since the PEI has positively charged mass amidogens, both the 2 mg/mL PG nanosheets and the 2 mg/mL TC/PG nanocomposites became positively charged, and their Zeta potentials were found to be 35.8 ± 0.7 mV and 20.3 ± 0.3 mV, respectively. These positive charges help the TC/PG nanocomposites to approach the bacterial cell membrane.

### 3.2. FTIR and Raman Analysis

**Figure 2:**
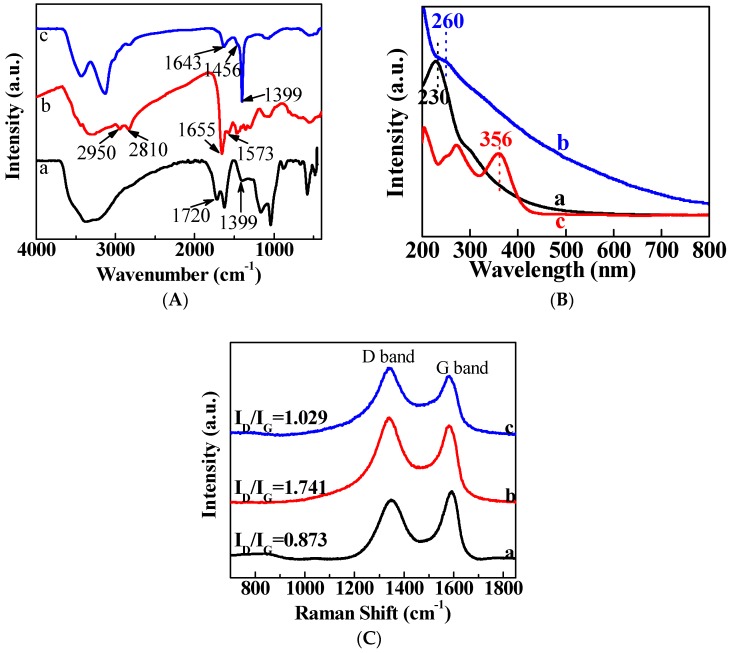
FTIR spectra (**A**), UV–vis absorption spectra (**B**), and Raman spectra (**C**) of GO nanosheets (**a**), PG nanosheets (**b**), and TC/PG (**c**) nanocomposites.

FTIR spectra of GO nanosheets, PG nanosheets, and TC/PG nanocomposites are displayed in Figure 2A. The characteristic bands at 1720 and 1399 cm^−1^ were assigned to the stretching vibrations of C=O and the bonding of C–OH of GO (curve a), respectively [1]. After PEI functionalization (curve b), the characteristic peak at 1720 cm^−1^ disappears. The peaks at 2950 and 2810 cm^−1^ were attributed to –CH_2_– stretching vibrations of the PEI. The peak located at 1655 cm^−1^ originated from C=O stretching vibrations of amide bonds [35,36]. Moreover, the peak located at 1573 cm^−1^ corresponded to the N–H bond of the primary amino acid of PEI [37]. Therefore, we can confirm that a covalent bond has formed between GO and PEI. Moreover, a hydrogen-bonding interaction between GO and PEI could also exist, since GO has a large number of oxygen-containing functional groups and PEI chains have many –NH_2_ groups. In the TC/PG nanocomposite spectrum (curve c), a new peak located at 1456 cm^−1^ can be seen that corresponds to the C=C vibration of the aromatic ring of TC [38]. Thus, we can confirm that the TC was successfully loaded onto the PG nanosheets.

Figure 2B shows the UV–vis absorption spectra of the GO nanosheets, the PG nanosheets, and the TC/PG nanocomposites, respectively. The spectrum for GO nanosheets (curve a) displays a sharp peak at 230 nm that corresponds to the electronic π−π* transitions of aromatic C−C bonds [39]. The spectrum for PG nanosheets (curve b) shows that the peak at 230 nm vanished and another peak appeared at 260 nm, which was attributed to the reduction ability of PEI that led to the formation of PEI–reduced graphene oxide (rGO) nanocomposites [40]. In the spectrum for the TC/PG nanocomposites, a new peak appeared at 356 nm that corresponded to the characteristic peak of TC [41]. Thus, we can verify that TCH was successfully loaded onto PG nanosheets.

Raman spectroscopy was employed in order to illustrate the structural changes in functionalized GO. Raman spectra of GO nanosheets, PG nanosheets, and TC/PG nanocomposites are displayed in Figure 2C. In the spectra for the GO nanosheets (curve a), the two bands located at 1580 cm^−1^ and 1352 cm^−1^ were denoted the D band and the G band, respectively. The intensity ratio between the D band and the G band (I_D_/I_G_) was determined to assess the surface defect density. Although both D and G bands exist in the Raman spectra of the PG nanosheets (curve b) and the TC/PG nanocomposites (curve c), their intensities have changed. The I_D_/I_G_ value of the GO nanosheets (curve a) was found to be 0.873. The I_D_/I_G_ of the PG nanosheets (curve b) increased to 1.741, which may be due to the increased number of defects in the lattices and stretching based on the graphene layers that occurred, in part, because of the formation of chemical bonds between GO and PEI. In the case of the TC/PG nanocomposites (curve c), the I_D_/I_G_ was lower (1.029) than that of the PG nanosheets, because the TC that was loaded onto the nanocomposite covered some of the defects on the PG’s surface.

### 3.3. TC Release Performance

**Figure 3:**
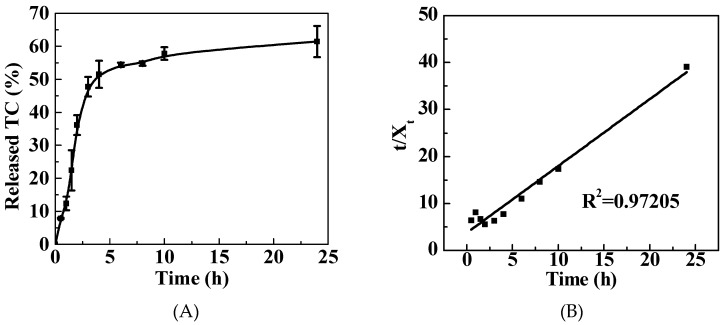
TC release behavior (**A**) and the release kinetic model (**B**) of the TC/PG nanocomposites.

The TC release profile is shown in Figure 3A. A rapid TC release behavior in the first 120 min was observed with about 52% of released TC, which is due to the ‘burst-release’ effect. Then, a controlled and sustained TC release behavior after 120 min was observed. The drug release mechanism was studied using mathematical modeling. A pseudo second order kinetic model was employed to illustrate the TC release mechanism. The correlation coefficient (R^2^) was used to identify the model that was suitable to describe the drug release mechanism. In this study, a pseudo second order kinetic model (Figure 3B) was considered to be a suitable model to illustrate the TC release behavior from the PG matrix, as the R^2^ was determined to be 0.97205. This result is consistent with that reported by Barahuie et al., who found that the same drug release mechanism was exhibited using GO as the drug carrier [10].

### 3.4. Antibacterial Performance

**Figure 4:**
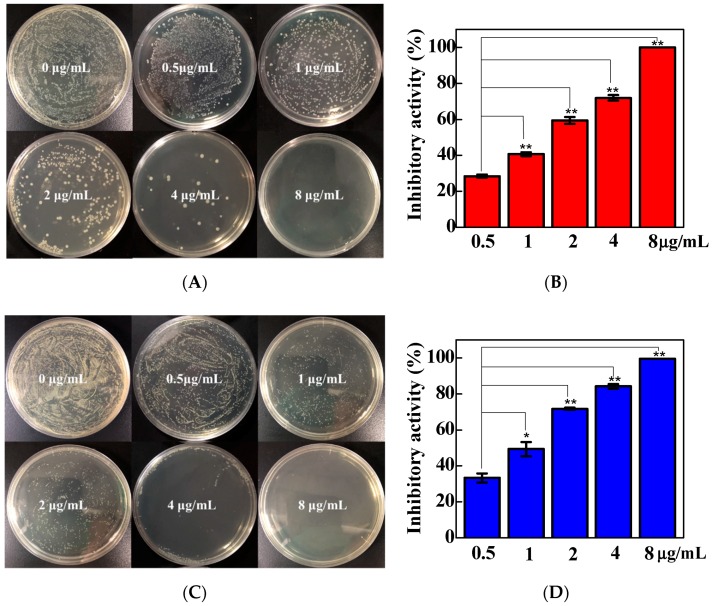
Images of *Escherichia coli* (**A**) and *Staphylococcus aureus* (**C**) on TSA plates after 24 h of incubation after treatment with different concentrations of TC/PG nanocomposites, and the corresponding inhibition activity against *E. coli* (**B**) and *S. aureus* (**D**).

Figure 4A,C show the numbers of bacterial colonies after treatment with different concentrations of TC/PG nanocomposites followed by dilution to 10^−3^ times the original concentration and culture on TSA plates for 24 h. A large number of *E. coli* colonies was observed on the plate without TC/PG nanocomposite treatment (Figure 4A). The number of *E. coli* colonies decreased as the concentration of the TC/PG nanocomposite increased. No *E. coli* colonies were detected on the TSA plates after treatment with the 8 ug/mL TC/PG nanocomposite. The calculated inhibition activity is shown in Figure 4B. The inhibition activity of the 0.5 ug/mL TC/PG nanocomposite against *E. coli* was 28.5%. The inhibition effect was enhanced as the concentration of the TC/PG nanocomposite increased. For the 8 ug/mL TC/PG nanocomposite, the inhibition activity reached 99.9%. Therefore, the TC/PG nanocomposites have a highly concentration-dependent inhibition effect on *E. coli*. The TC/PG nanocomposites showed similar inhibition behaviour against *S. aureus*. TC/PG nanocomposites have a highly concentration-dependent inhibition effect on *S. aureus*. In the case of the 8 ug/mL TC/PG nanocomposite, no *S. aureus* colonies were found on the plate (Figure 4C), and the inhibition activity reached 99.5% (Figure 4D). The TC/PG nanocomposites exhibit superior antibacterial activity against *E. coli* and *S. aureus.*

**Figure 5:**
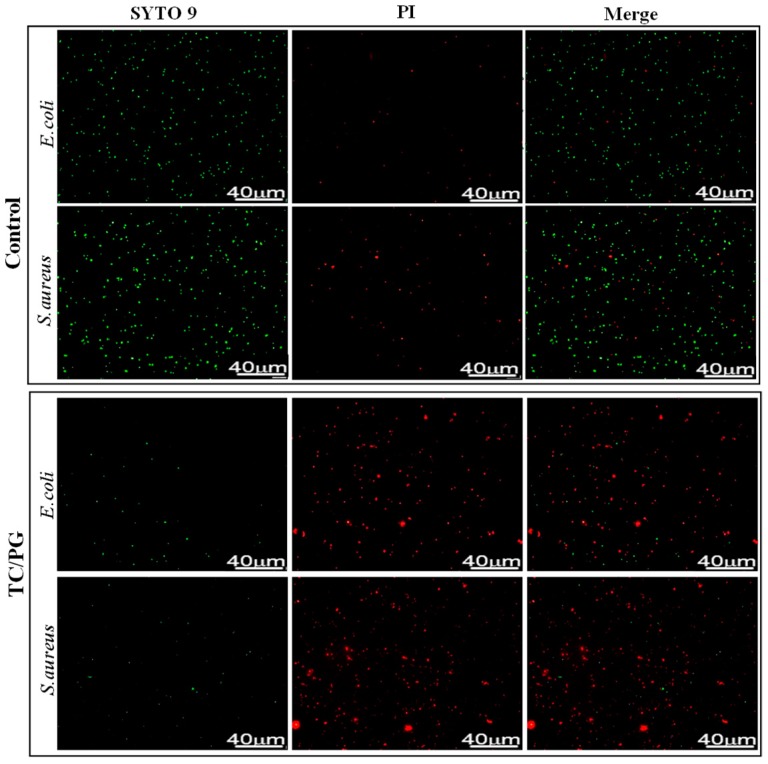
Images of the stained bacteria.

The antibacterial mechanism of TC/PG nanocomposites was studied by staining the treated bacteria using a LIVE/DEAD BacLight Bacterial Viability Kit (L13152). Strains, regardless of whether they are live with intact membranes or dead with damaged membranes, can be stained by SYTO 9, and exhibit fluorescent green. Dead strains can be stained by PI due to their damaged membranes, which emit red fluorescence. So, the number of dead or alive bacteria is represented by red or green fluorescent dots, respectively. Pictures of the stained cells are shown in Figure 5. For the control group, little red fluorescence is displayed, indicating that the untreated cells are alive. Accordingly, after treatment with TC/PG nanocomposites, almost all of tested cells exhibited fluorescent green. This result indicates that the cell membranes were damaged after being exposed to the TC/PG nanocomposites. In other words, *E. coli* and *S. aureus* cell death was induced via destruction of the integrity of the cell membrane by the TC/PG nanocomposites. This confirms the excellent antibacterial activity of the TC/PG nanocomposites.

The antibacterial activity of the fabricated TC/PG nanocomposites was evaluated by the disk diffusion method. The size of the inhibition zone directly reflects the bacterial inhibition ability since the inhibition zone is generated by the inhibition effect of the tested material on bacterial growth [42]. Images of the inhibition zone of the GO and the TC/PG nanocomposites are shown in Supplementary Figure S1. No noticeable inhibition zone can be found around the GO nanosheets, indicating that the GO nanosheets did not exhibit any inhibition through a leaching effect on *E. coli* and *S. aureus*. There are clear inhibition zones around the TC/PG nanocomposites, which exhibit a strong inhibition effect on the tested strains. This phenomenon is due to the TC that was loaded onto the nanocomposites, which can leach out from the TC/PG nanocomposites to inhibit the growth of the tested bacteria. The prepared TC/PG nanocomposites have an excellent inhibition effect.

**Figure 6:**
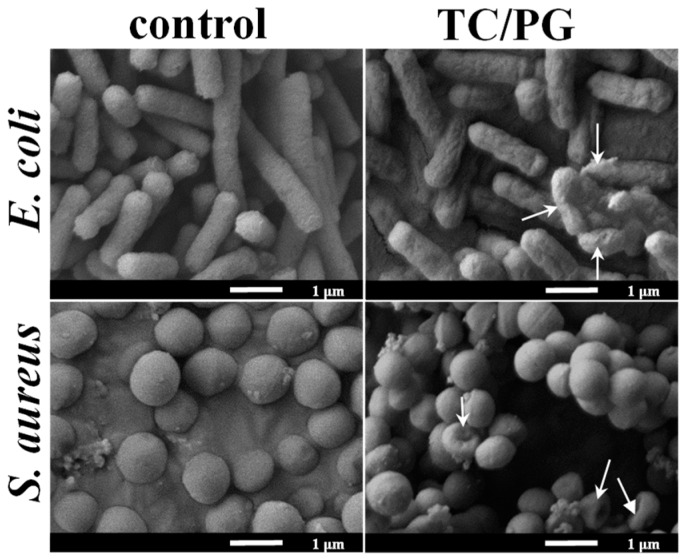
The morphologies after treatment with TC/PG nanocomposites. White arrows point to the damaged cells.

To further examine the antibacterial effect of the TC/PG nanocomposites, the integrity of the cell membrane after being treated with TC/PG nanocomposites was studied by SEM. As illustrated in Figure 6, native *E. coli* cells have a rod shape with intact and plump cell walls. The *E. coli* cells had wrinkled and damaged morphologies after incubation with the TC/PG nanocomposites. A similar result was obtained for *S. aureus*. Native *S. aureus* cells exhibit a plump and round shape with a smooth surface morphology. After being cultured with TC/PG nanocomposites, the shapes of *S. aureus* cells became distorted, and the cells exhibited wrinkled and damaged cell membranes with some intracellular inclusion leakage. So, the TC/PG nanocomposites demonstrate great antibacterial performance on *E. coli* and *S. aureus* through the destruction of cell structures with the compromise of the integrity of the bacterial cell membrane and the leakage of cytoplasm.

### 3.5. Protein Leakage

**Figure 7:**
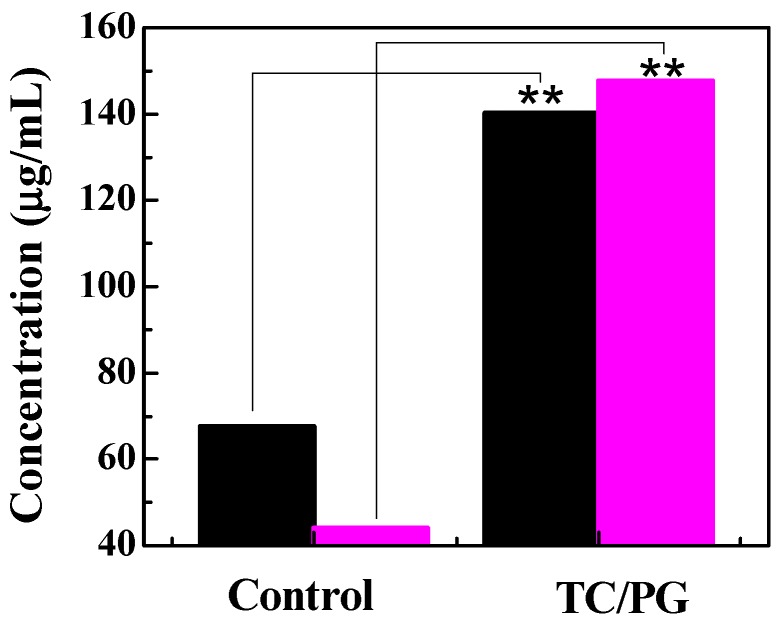
The protein leakage concentration in *E. coli* (black column) and *S. aureus* (pink column) suspensions treated with TC/PG nanocomposites.

The existence of protein in the bacterial suspension represents damage to bacterial cell membranes [43]. The amount of protein leakage was evaluated using a BCA Protein Assay Kit. The BCA protein assay forms a purple-colored and water-soluble BCA/copper complex, which exhibits an absorbance at 562 nm. The result is shown in Figure 7. The detected protein concentration without treatment was 67 µg/mL for *E. coli*. Some proteins were found to have leaked into the bacterial suspension for the control group, indicating that some membrane damage had occurred. However, a higher protein concentration (140 µg/mL) for the bacterial suspension treated with TC/PG nanocomposites was observed, suggesting that more leakage of cell content had happened. A lower amount of protein leakage, with a concentration of 44 µg/mL, was observed for untreated *S. aureus*. The detected protein concentration in *S. aureus* with TC/PG nanocomposite treatment was 148 µg/mL, which was more than 2 times than that of the control. This phenomenon was due to the enhanced bacterial membrane damage capability of the fabricated TC/PG nanocomposites. Moreover, the TC/PG nanocomposites exhibited a greater damaging effect on the cell membranes of *S. aureus* than *E. coli*. This is consistent with the results from the disk diffusion experiment.

### 3.6. Oxidative Stress-Mediated Antimicrobial Property

**Figure 8:**
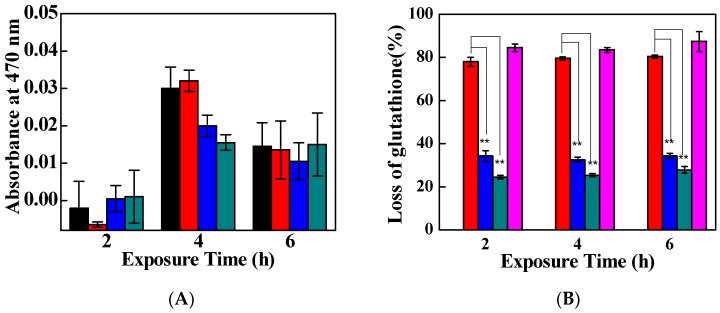
Oxidative stress mediated by the TC/PG nanocomposites. (**A**): reactive oxygen species (ROS)-dependent oxidative stress at exposure times of 2–6 h monitored using XTT tests. (**B**): Loss of glutathione after incubation with TC/PG nanocomposites for 2–6 h. Black columns represent the negative control, red columns represent GO nanosheets, blue columns represent PG nanosheets, and dark cyan columns represent TC/PG nanocomposites. H_2_O_2_ (the magenta column) was used as a positive control.

Physical interaction and oxidative stress formation are deemed to be responsible for the antibacterial performance of graphene-based materials [13]. GO nanosheets with sharp edges can puncture or penetrate into the cell membrane by direct contact with bacteria to disrupt their physical integrity, leading to the destruction of the bacterial cell membrane, intracellular component leakage, the uptake of membrane-impermeable dyes, and changes in transmembrane potential [17]. GO nanosheets can induce chemical stress in bacteria in order to generate oxidative stress and degrade the structure of cells regardless of whether any reactive oxygen species (ROS) are produced [44]. The ROS-dependent/independent oxidative stress pathways mediated by TC/PG nanocomposites were determined by the XTT method and an Ellman’s assay, respectively. The absorbance at 470 nm reflects the capacity for ROS production. No significant difference was found in the ROS production capacities among the tested samples and the control at 2 h, 4 h, or 6 h of incubation (Figure 8A). Moreover, only trace amounts of superoxide anions were formed by the TC/PG nanocomposites, since we barely detected any absorbance at 470 nm. In other words, the TC/PG nanocomposites did not show the ability to mediate ROS-dependent oxidative stress against bacteria due to their low production of superoxide anions.

The possibility for ROS-independent oxidative stress formed by TC/PG nanocomposites was evaluated using an Ellman’s assay. Elman’s reagent can quantitate thiols by forming a mixed disulfide that has a maximum absorption at 410 nm [45]. Glutathione (GSH) losses after incubation with TC/PG nanocomposites for 2–6 h are illustrated in Figure 8B. The negative control did not produce any oxidation of GSH. H_2_O_2_, acting as the positive control, caused a very high fraction of glutathione loss with incubation for 2 h, 4 h, and 6 h. The GO nanosheets also showed a great capability for GSH oxidation; the glutathione losses were found to be more than 78%. The TC/PG nanocomposites showed a reduced oxidation capacity of 24.4 ± 0.9%, 25.3 ± 0.7%, and 27.8 ± 1.5% for 2, 4, and 6 h of incubation, respectively. Thus, the fabricated TC/PG nanocomposites were indirectly shown to possess some GSH oxidation ability, which mediates ROS-independent oxidative stress toward bacteria.

In summary, the great bacterial inhibition behavior is due to the synergistic effect of the PG nanosheets and the TC. First, due to the existence of the high-density amine groups in the TC/PG nanocomposites, they can easily adsorb onto cell membranes with a negative charge via electrostatic interactions. Second, a physical interaction occurs upon direct contact with the bacteria and ROS-independent oxidative stress is generated that produces some oxidation capacity. Third and finally, TCH released from the TC/PG nanocomposites effectively restrains the growth of bacteria by inhibiting the synthesis of bacterial protein.

## 4. Conclusions

In this study, TC-loaded PG nanocomposites were constructed and successfully prepared. The developed TC/PG nanocomposites exhibit a slow and sustained TC release behavior, indicating that PG nanosheets can be utilized as a good TC carrier. Moreover, the TC/PG nanocomposites exhibit a great bacterial inhibition capacity towards *E. coli* and *S. aureus*. The antibacterial performance relies on the synergistic effect between TC and the PG nanosheets in the nanocomposite. These results clearly demonstrate that the prepared nanocomposites have great potential for application as an antibacterial agent.

## Supplementary Materials

The following are available online at https://www.mdpi.com/2079-4991/9/7/913/s1, Figure S1: Inhibition zone pictures of GO, TC/PG and TC against *E. coli* and *S. aureus*; Table S1: Diameters (mm) of Inhibition zone of GO, TC/PG and TC against *E. coli* and *S. aureus*; Figure S2: XRD spectra of GO and TC/PG nanocomposites.
