# Supplementary material for: Facile Construction of Functionalized GO Nanocomposites with Enhanced Antibacterial Activity
Source: Nanomaterials (Basel). 2019 Jun 26;9(7):913. doi: 10.3390/nano9070913 (PMC6669633; doi:10.3390/nano9070913)
Supplement: Supplementary file 1 [file nanomaterials-09-00913-s001.pdf]

Supporting Information

Facile Construction of Functionalized GO Nanocomposites with Enhanced Antibacterial Activity

Lei Jiang [1,2], Zhongjie Zhu [2], Yanyi Wen [2], Shan Ye [2], Chen Su [2], Rui Zhang [2,*], and Wei Shao [1,2,*]

[1] Jiangsu Co-Innovation Center of Efficient Processing and Utilization of Forest Resources, Nanjing Forestry University, Nanjing 210037, China; 15655679696@163.com (L.J.)

[2] College of Chemical Engineering, Nanjing Forestry University, Nanjing 210037, China; 13382367651@163.com (Z.Z.); wenyanyis@163.com (Y.W.); 13451867328@163.com (S.Y.); 13260866882@163.com (C.S.)

* Correspondence: zhangrui@njfu.edu.cn (R.Z.); w.shao@njfu.edu.cn (W.S.); Tel.: +86-25-85427024

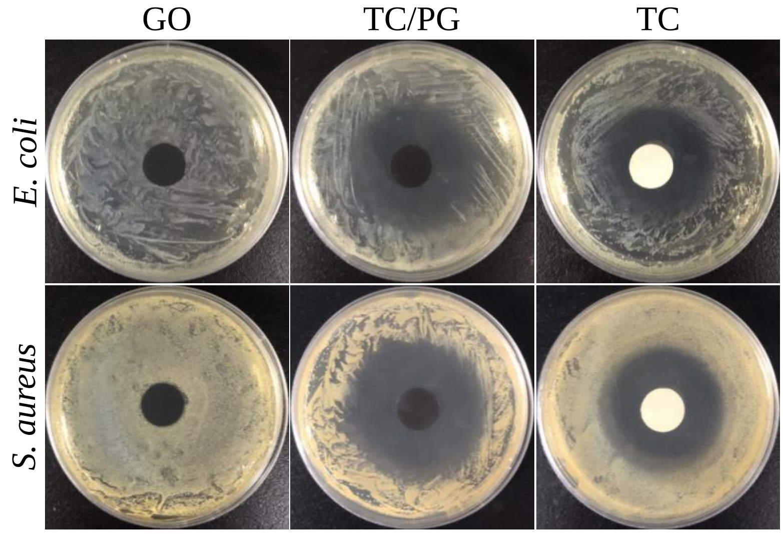

Figure S1. Inhibition zone pictures of GO, TC/PG and TC against *E. coli* and *S. aureus.*

Table S1. Diameters (mm) of Inhibition zone of GO, TC/PG and TC against *E. coli* and *S. aureus.*

	**GO**	**TC/PG**	**TC**
E. coli	-	29.5 ± 0.7	26.5 ± 0.7
S. aureus	-	32.5 ± 0.7	28.5 ± 0.7

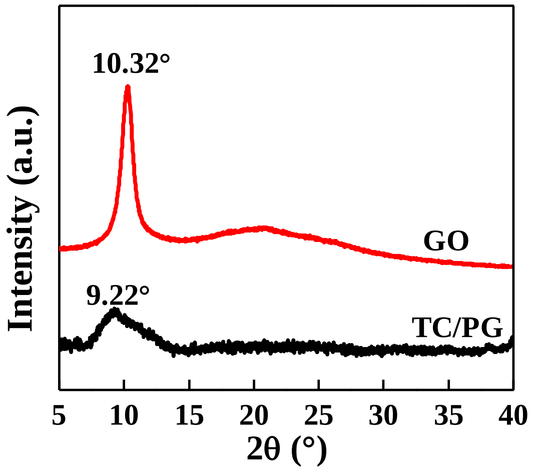

Figure S2. XRD spectra of GO and TC/PG nanocomposites.

The XRD patterns of GO and TC/PG nanocomposites are listed in Figure S2. The characteristic diffraction peak of GO was at 10.32° and the interlayer distance was calculated to be 0.88 nm according to the Bragg equation. For TC/PG nanocomposite, the interlayer distance changed to 0.98 nm because the diffraction peak shifted to 9.22°. The increase in the interlayer distance confirmed the successful loading of TC onto PG nanosheets.
